# Demographic, clinical, and laboratory parameters of cystic fibrosis during the last two decades: a comparative analysis

**DOI:** 10.1186/1471-2466-15-3

**Published:** 2015-01-15

**Authors:** Fernando Augusto de Lima Marson, Tais Daiene Russo Hortencio, Katia Cristina Alberto Aguiar, Jose Dirceu Ribeiro, CYFIUC Group

**Affiliations:** Departments of Pediatrics, State University of Campinas - Unicamp, Faculty of Medical Sciences, Campinas, Brazil; Departments of Medical Genetics, State University of Campinas - Unicamp, Faculty of Medical Sciences, Campinas, Brazil

**Keywords:** Cystic fibrosis, Epidemiology, Lung disease

## Abstract

**Background:**

In recent years, patients with cystic fibrosis (CF) have tended to experience a longer life expectancy and higher quality of life. In this context, the aim of the present study was to evaluate and compare the demographic, clinical, and laboratory markers of patients with CF during the last two decades at a CF referral center.

**Methods:**

A retrospective study of the demographic, clinical, and laboratory markers for CF treatment at a CF referral center was performed during two decades: 2000 (DI, 1990–2000, n = 104 patients) and 2010 (DII, 2000–2010, n = 181 patients).

**Results:**

The following variables were less common in DI than in DII: (i) pancreatic insufficiency, (ii) meconium ileus, (iii) diabetes mellitus, (iv) *Burkholderia cepacia* colonization, (v) moderate and severe Shwachman-Kulczycki score (SKS), (vi) F508del mutation screening, (vii) patients without an identified *CFTR* mutation (class IV, V, or VI mutation), (viii) patients above the 10th percentile for weight and height, (ix) restrictive lung disease, and (x) older patients (p < 0.01). The following variables were more common in DI than in DII: (i) excellent and good SKS, (ii) F508del heterozygous status, (iii) colonization by mucoid and nonmucoid *Pseudomonas aeruginosa*, (iv) obstructive lung disease, and (v) minimal time for CF diagnosis (p < 0.01).

**Conclusion:**

Clinical outcomes differed between the two decades. Demographic, clinical, and laboratory markers in patients with CF are useful tools and should be encouraged in CF referral centers to determine the results of CF management and treatment, enabling a better understanding of this disease and its clinical evolution. Early diagnosis and management of CF will improve patients’ quality of life and life expectancy until personalized drug therapy is possible for all patients with CF.

## Background

The advances in knowledge of cystic fibrosis (CF: #219700) have been striking in recent decades. Identification of the cystic fibrosis transmembrane regulator gene (*CFTR*) (region 7q3.11) [1–3], with nearly 2,000 mutations identified in 2014 [4–6], has provided the knowledge base for the genotypic and phenotypic characteristics of CF. *CFTR* mutations lead to the absence or dysfunction (quantitative and/or qualitative) of the CFTR protein, altering chloride transport at the cell surface. This causes a compensatory influx of sodium to maintain electroneutrality and a consequent influx of water, extracellular dehydration, and formation of thick mucus in the airways [7]. Classic CF is characterized by pulmonary and gastrointestinal symptoms in the first few months of life [8].

The clinical manifestations of CF are variable, even among patients with the same *CFTR* mutation [9, 10]. Advances in CF treatment in the last several years are related to therapies aimed at improving the quality of life and management of the disease. These therapies include inhaled drugs, respiratory therapy, individualized outpatient care, treatment of comorbidities, genetic counseling, dietary adaptation of pancreatic enzymes, nutritional supplements, neonatal screening by immunoreactive trypsinogen (IRT), antibiotic therapy, lung transplantation, and, more recently, personalized drug therapy [i.e., ivacaftor for *G551D* (rs75527207, c.1652G > A) mutation and eight other mutations approved by the US Food and Drug Administration] [11, 12].

In developing countries, recent studies on CF are scarce and a poor understanding of patient management and CF progression during follow-up care exists in referral centers. In this context, the aim of the present study was to evaluate and compare the demographic, clinical, and laboratory markers in patients with CF treated at a referral center in the last decade of the 20th century (DI) and the first decade of the 21st century (DII).

## Methods

A retrospective study in a CF referral center was performed. Demographic, clinical, and laboratory data were obtained from 104 patients with CF seen in the last decade of the 20th century, from 1990 (time zero) to 2000 (DI); all data were collected from the patients’ medical records in the year 2000 [13]. The same data were obtained from 181 patients with CF treated in the first decade of the present century, from 2001 (time zero) to 2010 (DII); all data were collected from the patients’ medical records in the year 2011. The number of patients with CF enrolled in both decades equaled the total number of patients with CF followed at the referral center in the stipulated timeframe. The diagnosis of CF was confirmed by two sweat tests with chloride concentrations of >60 mEq/L (gold standard diagnostic tool) and/or identification of two *CFTR* mutations. Patients with CF who had undergone lung transplantation were not enrolled.

### Demographic, clinical, and laboratory markers

The demographic, clinical, and laboratory variables analyzed in this study were sex (male/female), ethnicity (Caucasian or non-Caucasian), age, age range, number of deaths, clinical manifestations (respiratory and digestive), age at diagnosis, comorbidities [pancreatic insufficiency (PI), meconium ileus (MI), and diabetes mellitus (DM)], nutritional status as determined by weight and height on a growth curve (weight and height below the 10th percentile), oxygen saturation (SpO_2_) (>95%, 91%–95%, or <91%), sweat chloride level, microorganisms in the sputum (*Staphylococcus aureus*, mucoid and nonmucoid *Pseudomonas aeruginosa*, and *Burkholderia cepacia*), spirometry findings (normal, restrictive lung disease, obstructive lung disease, or mixed respiratory disorder) [14], genetic screening for the *CFTR* mutations [F508del (rs113993960, c.1521_1523delCTT), G542X (rs113993959, c.1624G > T), N1303K (rs80034486, c.3909C > G), G551D, R553X (rs74597325, c.1657C > T), and W1282X (rs77010898, c.3846G > A)], Shwachman-Kulczycki score (SKS) (excellent or good, mild, or moderate or severe) [15], and fecal fat.

### Spirometry

Spirometric testing was performed according to the following American Thoracic Society standards [14]:

(i). Obstructive lung disease: less than the 5th percentile of the ratio of the predicted forced expiratory volume in the first second to the forced vital capacity (FEV_1_/FVC). Reduction of flow in a patient with low lung volume is not specific to small airway disease. Concomitant decreases in FEV_1_ and FVC are commonly caused by poor effort, but can rarely reflect airflow obstruction. Confirmation of airway obstruction requires lung volume measurement.
(ii). Restrictive ventilatory disturbance: total lung capacity below the 5th percentile of the predicted value. Reduced FVC does not prove the presence of a restrictive lung defect, but is suggestive of pulmonary restriction when the FEV_1_/FVC is normal or increased.
(iii). Mixed respiratory disorder: FEV_1_/FVC and lung capacity below the 5th percentile of the predicted value.

Spirometric testing was performed in patients >7 years of age using a CPFS/D spirometer (Medical Graphics Co., Saint Paul, MN, USA). The data were recorded by PF BREEZE software vs 3.8B for Windows 95/98/NT (Medical Graphics Co.) [14].

### SKS analysis

The SKS was determined by two previously trained professionals, and in case of disagreement, a third evaluator was considered [15].

### Sputum collection

The patients’ sputum was collected by spontaneous sputum sampling, induction by physiotherapy maneuvers, or collection of oropharyngeal swabs for analysis of the microorganism colonizing or infecting the airways. Examinations were performed in the Pathology Laboratory at the Clinical Hospital of the State University of Campinas.

IRT was implemented in our center in 2010. Therefore, the comparative analysis of the two decades did not include patients with CF who underwent neonatal screening.

### Statistical analysis

The Statistical Package for the Social Sciences v.21.0 (SPSS, Inc., Chicago, IL, USA) was used for statistical analysis [16]. For descriptive analysis, we used mean, median, and standard deviation for continuous variables and absolute frequency (percentage) for categorical variables. Fisher’s exact test and the χ^2^ test were performed to compare the groups using categorical variables. In cases of differences in the data between the periods analyzed, the odds ratio (OR) and 95% confidence interval (95% CI) were calculated. The alpha value was 0.05. The Ethics Committee in Research of the State University of Campinas (#508/2008) approved this study.

## Results

Table 1 compares the demographic, clinical, and laboratory variables of the patients with CF enrolled in this study in the two above-described decades.

**Table 1:** **Comparison of data (demographic, clinical, and laboratory markers) of patients with cystic fibrosis from a Brazilian referral center during the decades of 1990 to 2000 and 2000 to 2010**

Demographic and clinical markers	Category	DI (1990–2000)	DII (2000–2010)	p
Number of patients		104	181	
Age		Mean: 10 years 9 months ± 6.33 months	Mean :16 years 7 months ± 13.16 months	**<0.001**
		Median: 9 years	Median: 12 years 10 months	
		Range: 11 months to 31 years 2 months	Range: 6 months to 73 years 10 months	
Sex	Male	53.8%	49.7%	0.539
	Female	46.2%	50.3%	
Ethnicity	Caucasian	93.3%	92.0%	0.818
	NonCaucasian	6.7%	8.0%	
Consanguineous parents		6.2%	1.1%	0.054
Manifestation	Respiratory	89.4%	91.7%	0.528
	Digestive	59.6%	83.3%	**<0.001**
Onset of symptoms		Mean: 16 months	Mean: 91.75 months	**<0.001**
		Median: 3 months	Median: 3 months	
		Range: 0–20 years	Range: 0–60 years	
Age at diagnosis		Mean: 4 years 2 months	Mean: 2 years 10 months	**<0.001**
		Median: 2 years 4 months	Median: 2 years	
		Range: 0 to 29 years 11 months	Range: 0–60 years	
Meconium ileus		5.8%	15.0%	**0.021**
Diabetes mellitus		4.8%	18.5%	**0.001**
Nutritional status	Weight below 10th percentile	69.9%	35.71%	**<0.001**
	Height below 10th percentile	56.6%	40.82%	**0.025**
SpO_2_	>95%	59.5%	55.5%	0.713
	91%–95%	32.9%	34.7%	
	<91%	7.6%	9.8%	
Sweat test	<60 mEq/L*	10.6%	–	
	60–100 mEq/L	28.8%	40.51%	
	>100 mEq/L	60.6%	59.49%	
Bacteria	*Staphylococcus aureus*	80.2%	78.5%	0.880
	*Pseudomonas aeruginosa*	76.0%	55.8%	**0.001**
	Mucoid *P. aeruginosa*	53.1%	42.0%	0.085
	*Burkholderia cepacia*	5.2%	14.4%	**0.016**
	Mucoid and nonmucoid *P. aeruginosa*	51.0%	21.85%	**<0.001**
Spirometry	Normal	27.3%	34.4%	**<0.001**
	Restrictive ventilatory disorder	18.2%	48.9%	
	Obstructive lung disorder	25.4%	14.5%	
	Mixed respiratory disorder	29.1%	2.3%	
*CFTR* mutation	F508del homozygotes	18.75%	26.5%	**<0.001**
	F508del heterozygotes	62.5%	22.7%	
	G542X	4.17%	6.45%	
	N1303K	2.08%	1.1%	
	G551D	1.04%	–	
	R553X	0.52%	0.3%	
	W1282X	0.52%	–	
Shwachman-Kulczycki score	Excellent or good	57.8%	36.2%	**0.005**
	Mild	26.5%	36.2%	
	Moderate or severe	15.7%	27.6%	
Deaths		18	31	1
Fecal balance		67.9%	80.0%	**0.031**

DI – period from 1990 to 2000; DII – period from 2000 to 2010; SpO_2_ – transcutaneous hemoglobin saturation by oxygen; p – p-value. Statistical analysis was performed by the χ^2^ test. Statistically significant values are indicated by bold font. *Patients with two identified *CFTR* mutations.

The following markers showed an association between the two decades: digestive symptoms, comorbidities, height and weight, microorganisms, spirometry, SKS, F508del screening, and age range (Tables 2, 3, 4 and 5).

**Figure 1:**
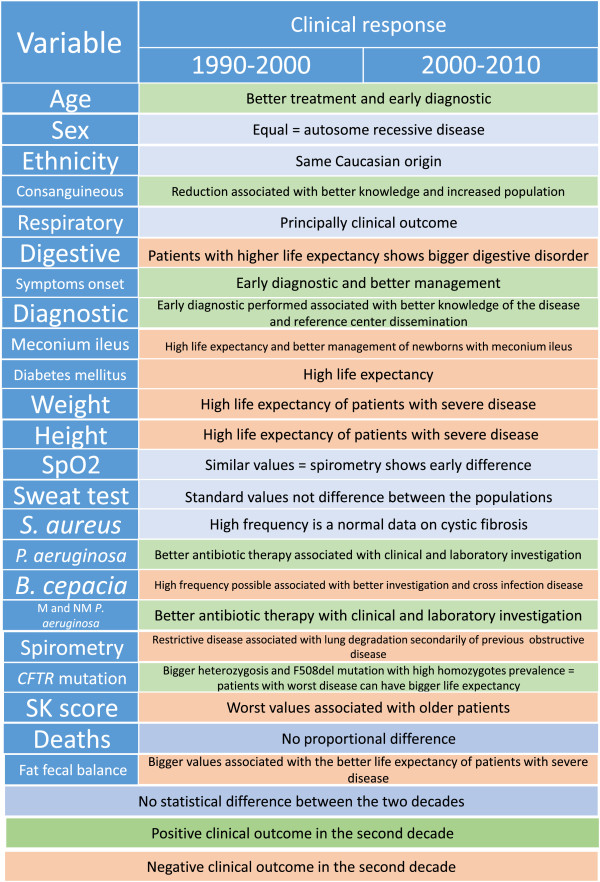
**Clinical outcome variables the between two decades of data for patients with cystic fibrosis from a Brazilian referral center.** SpO_2_, − transcutaneous hemoglobin saturation by oxygen; SKS, − Shwachman-Kulczycki score; M and NM, − mucoid and nonmucoid.

An overview of all data analyzed is shown in Figure 1. Comparison of DI with DII revealed the following in DI:

**Table 2:** **Presence of digestive symptoms, comorbidities, and percentiles for weight and height in patients with cystic fibrosis in a Brazilian referral center during the decades of 1990 to 2000 and 2000 to 2010**

Decade	Onset of digestive symptoms			p-value	OR	95% CI
	Presence	Absence	Total			
1990–2000	62 (59.6%)	42 (40.4%)	104	**<0.001**	**0.295**	**0.17–0.51**
2000–2010	151 (83.4%)	30 (16.6%)	181		1	–
Decade	Meconium ileus			p	OR	95%CI
	Presence	Absence	Total			
1990–2000	6 (5.8%)	98 (94.2%)	104	**0.021**	**0.35**	**0.11–0.91**
2000–2010	27 (14.9%)		181		1	–
Decade	Diabetes mellitus			p	OR	95%CI
	Presence	Absence	Total			
1990–2000	5 (4.8%)	99 (95.2%)	104	**0.001**	**0.228**	**0.07–0.61**
2000–2010	33 (18.2%)	148 (81.8%)	181		1	–
Decade	Fecal fat balance			p	OR	95%CI
	Presence	Absence	Total			
1990–2000	71 (68.3%)	33 (31.7%)	104	**0.031**	**0.534**	**(0.31–0.93)**
2000–2010	145 (80.1%)	36 (24.2%)	181		1	–
Decade	Weight percentile			p	OR	95%CI
	P < 10	P ≥ 10	Total			
1990–2000	73 (70.2%)	31 (29.8%)	104	**<0.001**	**4.206**	**2.34–7.67**
2000–2010	35 (35.9%)	63 (64.1%)	98		1	–
Decade	Height percentile			p	OR	95%CI
	P < 10	P ≥ 10	Total			
1990–2000	59 (56.7%)	45 (43.3%)	104	**0.025**	**1.895**	**1.08–3.34**
2000–2010	40 (40.9%)	58 (59.1%)	98		1	–

P – percentile; p – p-value; OR – odds ratio; CI – confidence interval. Statistical analysis was performed by the χ^2^ test. Statistically significant values are indicated by bold font.

**Table 3:** **Presence of bacteria in sputum samples of patients with cystic fibrosis in a Brazilian referral center during the decades of 1990 to 2000 and 2000 to 2010**

Decade	***Pseudomonas aeruginosa*** *			p-value	OR	95% CI
	Presence	Absence	Total			
1990–2000	79 (76.0%)	25 (24.0%)	104	**0.001**	**2.495**	**1.42–4.48**
2000–2010	101 (55.8%)	80 (44.2%)	181		1	–
Decade	*Burkholderia cepacia*			p-value	OR	95%CI
	Presence	Absence	Total			
1990–2000	5 (4.8%)	99 (95.2%)	104	**0.016**	**0.302**	**0.09–0.84**
2000–2010	26 (14.4%)	155 (85.6%)	181		1	–
Decade	Mucoid and nonmucoid *P. aeruginosa* ^#^			p-value	OR	95%CI
	Presence	Absence	Total			
1990–2000	53 (51.0%)	51 (49.0%)	104	**<0.001**	**3.645**	**2.11–6.38**
2000–2010	40 (22.1%)	141 (77.9%)	181		1	–

OR – odds ratio; CI – confidence interval. Statistical analysis was performed by the χ^2^ test. Statistically significant values are indicated by bold font. *Analysis considering the presence of nonmucoid *P. aeruginosa* isolated in culture. ^#^Analysis considering the simultaneous presence of mucoid and nonmucoid *P. aeruginosa*.

**Table 4:** **Spirometry, Shwachman-Kulczycki scores, and prevalence of F508del mutation in patients with cystic fibrosis in a Brazilian referral center during the decades of 1990 to 2000 and 2000 to 2010**

Decade	Spirometry								Total
	ORD	OR (95% CI)	MRD	OR (95% CI)	RVD	OR (95% CI)	Normal	OR (95% CI)	
1990–2000	14 (25.0%)	2.058 (0.87–4.79)	16 (29.8%)	**17.58 (4.70–98.97)**	10 (18.3%)	**0.231 (0.10–0.51)**	15 (26.9%)	0.719 (0.33–1.50)	55
2000–2010	19 (14.2%)	1	3 (2.2%)	1	66 (49.3%)	1	46 (34.3%)	1	134
**Decade**	**Clinical score**								**Total**
	**Moderate/Severe**	**OR (95% CI)**		**Mild**	**OR (95% CI)**	**Excellent/Good**		**OR (95% CI)**	
1990–2000	13 (15.4%)	**0.491 (0.24–0.96)**		22 (26.9%)	0.635 (0.34–1.18)	48 (57.7%)		**2.403 (1.40–4.16)**	83
2000–2010	44 (27.5%)	1		58 (36.3%)	1	58 (36.3%)		1	160
**Decade**	**F508del mutation genotype**								**Total**
	**Homozygotes**	**OR (95% CI)**		**Heterozygotes**	**OR (95% CI)**	**Without F508del**		**OR (95% CI)**	
1990–2000	19 (20.2%)	0.647 (0.34–1.21)		59 (61.5%)	**5.445 (3.09–9.74)**	18 (18.3%)		**0.235 (0.13–0.42)**	96
2000–2010	50 (27.6%)	1		41 (22.7%)	1	90 (49.7%)		1	181

OR – odds ratio; CI – confidence interval; ORD – obstructive respiratory disorder; MRD – mixed respiratory disorder; RVD – restrictive ventilatory disorder. Statistical analysis was performed by the χ^2^ test. Statistically significant values are indicated by bold text.

**Table 5:** **Analysis of age of patients with cystic fibrosis in a Brazilian referral center during the decades of 1990 to 2000 and 2000 to 2010**

Age groups	Decades		p-value	OR (95% CI)
	1990–2000	2000–2010		
Newborn	0 (0.0%)	0 (0.0%)	<0.001	-
Infant	6 (7.0%)	6 (3.4%)		2.131 (0.63–7.19)
Preschool	25 (29.1%)	24 (13.6%)		**2.602 (1.38–4.94)**
Academic	18 (20.9%)	52 (29.4%)		0.636 (0.35–1.17)
Adolescent	27 (31.4%)	40 (22.6%)		1.567 (0.88–2.79)
Adult	10 (11.6%)	55 (31.0%)		**0.293 (0.13–0.60)**

OR – odds ratio; CI – confidence interval. Statistical analysis was performed by the χ^2^ test. Statistically significant values are indicated by bold text.

(i). fewer patients with digestive disease (OR = 0.295, 95% CI = 0.17–0.51);
(ii). fewer patients with MI (OR = 0.35, 95% CI = 0.11–0.91) and DM (OR = 0.228, 95% CI = 0.07–0.61);
(iii). fewer patients with an altered fat balance (OR = 0.534, 95% CI = 0.31–0.93);
(iv). more patients below the 10th percentile for weight (OR = 4.21, 95% CI = 2.34–7.67) and height (OR = 1.895, 95% CI = 1.08–3.34);
(v). a higher frequency of *P. aeruginosa* (OR = 2.495, 95% CI = 1.42–4.48) and concurrent mucoid and nonmucoid *P. aeruginosa* (OR =3.65, 95% CI = 2.11–6.38);
(vi). a lower frequency of *B. cepacia* (OR = 0.302, 95%CI = 0.09–0.84);
(vii). a higher frequency of mixed respiratory disorders (OR = 17.58, 95% CI = 4.70–98.87) and a lower frequency of restrictive respiratory disorders (OR = 0.231, 95% CI = 0.09–0.51);
(viii). a lower frequency of moderate and severe SKS (OR = 0.491, 95% CI = 0.24–0.96) and a higher frequency of excellent and good SKS (OR = 2.403; 95% CI = 1.40–4.16);
(ix). for F508del a heterozygosity mutation was more frequent (OR = 5.445, 95% CI = 3.09–9.74) in CF patients with two *CFTR* mutations screened and less frequent for patients without an identified *CFTR* mutation (OR = 0.235, 95% CI = 0.13–0.42); and
(x). a higher frequency of patients in preschool (OR = 2.602, 95% CI = 1.38–4.94) and fewer adult patients (OR = 0.293, 95% CI = 0.13–0.60).

**Figure 2:**
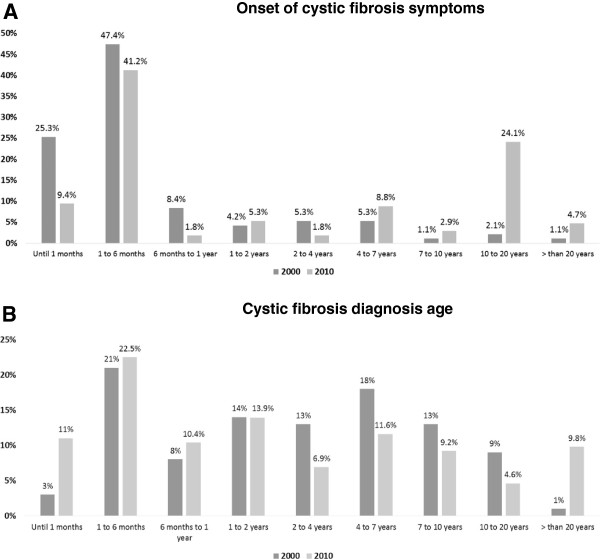
**Distribution of patients with cystic fibrosis according to ages at onset of symptoms and diagnosis. A**. Comparison of the distribution of patients with cystic fibrosis according to age at onset of symptoms between decades I (n = 95) and II (n = 181). **B**. Comparison of distribution of patients with cystic fibrosis according to age at diagnosis between decades I (n = 100) and II (n = 180).

Figure 2 shows the age at onset of clinical symptoms (Figure 2A) and at diagnosis (Figure 2B).

## Discussion

Evolutionary studies of the clinical and laboratory characteristics of patients with CF have been performed by many organizations worldwide. The Cystic Fibrosis Foundation [17] and the Cystic Fibrosis Trust [18] show the annual records of patients and compare the clinical outcomes in sequential decades. These data allow for appropriate actions by healthcare institutions and government and generate prospects for laboratory and clinical research on CF. However; few studies in Brazil have reported the characteristics of CF annually or in recent decades.

### Sex, ethnicity, and consanguinity

The sex prevalence was equal between the two decades. The disease severity and energy demand are greater both in patients with CF and during puberty, which may result in an increase in the long-term incidence of men with CF [19]. However, this finding was not observed in either DI or DII.

Another finding in this study relates to the prevalence of Caucasian patients with CF. The CF referral center considered is the same in two decades, and the enrolled population comprised patients from the same region; the patients were predominantly Caucasian. In this context, summing the Caucasian prevalence and CF etiology, which is intrinsically of Caucasian patients, values of >90% for the Caucasian patients in the two decades was expected.

The prevalence of consanguineous marriage was low at nearly 5%. As a monogenic recessive disease, children of consanguineous parents have a higher risk of developing CF. The public health policy in Brazil helps to lower the risks associated with consanguineous marriages by educating the population about these risks. This policy resulted in a lower number of patients with CF with consanguineous parents in DII than in DI.

### Patient age

Patients in DII were older than those in DI. Age is a factor associated with SKS variability [20] because CF lung disease is associated with a decline in lung function secondary to architectural deterioration of the airways caused by the cycle of inflammation/infection with chronic exacerbations due to bacterial infections [21]. Older patients constitute a major proportion of the patients seen at CF centers, and older age can be associated with variations in CF diagnosis and treatment [22].

### Digestive disease, PI, and fecal balance

An important finding is that the digestive symptoms exhibited the same frequency between the two decades. Digestive symptoms are often the first clinical marker of classic CF and are reported in 95% of patients with classic CF. Because no change in prevalence was observed, this marker continues to be a target for first-line treatment of patients with CF. After initial treatment, the severity of symptoms and progression of lung disease decrease.

The specific digestive symptoms associated with the severity of CF are PI, DM, and MI. Among these digestive symptoms, the principal marker of CF is PI. A diagnosis of PI was more prevalent in DI than in DII in this study. PI is principally associated with severe *CFTR* mutations and, secondarily, with modifier genes [23]. PI screening was performed by fecal fat and/or fecal elastase testing. Because no change in the diligence of PI screening occurred in our center during the two decades, we believe that the decreased frequency of PI with time can be explained by the inclusion of patients with mild CF.

### Diagnosis and nutrition status

Patients with CF have better clinical outcomes when diagnosed earlier [24]. This fact was highlighted in the comparison of DI and DII; both earlier diagnosis and improved clinical outcomes were noted in DII. In Brazil, the creation of the Brazilian Cystic Fibrosis Study Group and the establishment of referral centers have favored wider dissemination of CF screening guidelines. These facts contributed to the shorter diagnosis time in DII. We found that in patients with severe CR, the diagnosis time did not differ between DI and DII. For patients with less severe CF, however, the diagnosis time was shorter in DII than in DI (this was only seen as a difference between the mean diagnosis times, not the median).

Early diagnosis and IRT screening are associated with each other in patients with CF [25]. In the two decades of the present study, however, IRT screening was not used in our center. IRT screening was implemented in our state at the end of the second decade (2010) and in the entire country in 2014. Thus, we hope that in the next decade, patients with CF will receive a diagnosis in the first few months of life and that the clinical features of CF will continue to improve with earlier intervention.

Patients with CF who maintain normal growth in the first 2 years of diagnosis show better lung function, less coughing, and better chest radiographs at 6 years of age. The benefits of IRT screening for pulmonary status at 6 years of age depend on the initial nutritional status and are strongly associated with the need for comprehensive and consistent treatment implemented immediately after diagnosis [26, 27]. In the present study, earlier diagnosis in DII seemed to influence the nutritional status. There were fewer patients with CF below the 10th percentile for weight and height.

### Comorbidities: DM and MI

The prevalence of DM was 4.8% in DI and 18.5% in DII. This comorbidity is related to increased patient age, the arising of modifier genes [28] secondary to the inflammatory process, and consecutive destruction of the exocrine pancreas [29]. The severity of CF was greater in female than in male patients, especially during adolescence [30]. Patients with concurrent CF and DM have severe pulmonary disease with a higher prevalence of pathogens in the sputum, malnutrition, an increased incidence of liver disease, and increased mortality rates [30]. The recent increase in life expectancy for patients with CF has caused an increase in the prevalence of DM, and the mortality rates for these patients remain high [31].

The prevalence of MI was lower in DI than in DII; the prevalence in each decade was close to that cited in the literature (14% and 11%, respectively) [32]. This probably occurred because in DI, patients with concurrent CF and MI died in the first year of life, before the diagnosis of CF was confirmed [13, 33].

### Spirometry and lung disease

One of the most important markers of CF severity is the decline in spirometry values throughout life, as more than 90% of patients with CF die of lung disease. This decline in lung function varies with the presence of *CFTR* mutation, age at diagnosis, presence of PI, environmental pollution, nutritional status, adherence to treatment, comorbidities (DM, depression, osteopenia, and chronic infection), and sex. Airway changes caused by CF first affect the small airways, followed by the larger airways and finally the pulmonary parenchyma. The instruments used to measure pulmonary function have different sensitivities and specificities. However, spirometry is widely used to document longitudinal changes because it is both inexpensive and noninvasive. A reduction in the incidence of obstructive lung disease and an increase in the incidence of restrictive disorders was observed as time progressed from DI to DII. CF is primarily an obstructive lung disease, as observed in DI [34], while restrictive disorders were more prevalent in DII. Patients with class I, II, and III *CFTR* mutations have a worse prognosis; however, with aggressive treatment they can experience prolonged survival despite lower lung function. No difference was observed in the SpO_2_ in this study.

### Sweat chloride concentration

Measurement of the sweat chloride concentration by the Gibson and Cooke method [35] is the gold standard for a diagnosis of CF. Values of >60 mEq/L are indicative of CF. In DI, 10.6%, 28.8%, and 60.6% of patients had a sweat chloride concentration of 40 to 60, 60 to 100, and >100 mEq/L, respectively. In DII, all patients with CF had sweat chloride concentrations of >60 mEq/L. Patients with sweat chloride concentrations of <60 mEq/L in DI had other diagnostic criteria for CF such as respiratory and digestive symptoms compatible with CF, colonization by *P. aeruginosa*, and, principally, F508del mutation.

A difficult clinical problem is the assessment of individuals with signs and symptoms of CF but borderline sweat chloride concentrations. The diagnosis of CF can be clarified by evaporimetry, electrophysiology of epithelial tissues (measurement of the nasal potential difference and evaluation of the digestive epithelium using rectal biopsy or an Ussing chamber), and *CFTR* mutation screening in patients with signs and symptoms of CF, but with normal sweat test results [36–38].

### Microorganisms

In patients with CF, morbidity and mortality are associated with chronic bronchial infection by various pathogens [36]. Recent evidence suggests that the presence of such microorganisms along with opportunistic pathogens can affect the infection course and outcome [39]. No changes in the microbiologic profile of airway secretions in patients with nonmucoid and mucoid *P. aeruginosa* infection were observed between DI and DII. The main factors associated with chronic colonization or infection of airways by these bacteria in patients with CF are environmental contamination, rigorous treatment for the first colonization, screening diligence, increasing age, and genetic modulation [40, 41]. There was a higher frequency of *B. cepacia* isolation in sputum from the patients in DII. This finding may suggest that the environmental prophylaxis adopted in our center is not appropriate because it indicates cross-contamination among patients. Additionally, this increase may be due to the use of better screening tools in our diagnostic routine (specific polymerase chain reaction for *B. cepacia*). These facts may also account for the increased frequency of *Achromobacter xylosoxidans* isolation in DII. Thus, increasing patient age and diligence for *B. cepacia* identification by specific means must have been responsible for the higher rates of colonization and infection in DII.

### *CFTR*genotype

Currently, patients with CF can be divided into two groups using IRT screening: those with classic CF with class I, II, and III mutations who reach adulthood because they have received pediatric care, and those with CF caused by class IV, V, and VI mutations whose symptoms began in adolescence or adulthood.

Physicians who care for adults must consider these two groups of patients with CF because they have different genotypes and phenotypes. Future studies should assess and share the developments and clinical and laboratory characteristics of these groups as patients with typical and atypical CF. It is expected that patients with classic CF will develop impaired lung function in childhood and exhibit a serious decline in function until adulthood, while the same does not happen for patients with CF with mild mutations, in whom lung function values may be normal or near normal and who exhibit only a mild to moderate decline in function over their lifetime.

The most frequent *CFTR* mutations in this Brazilian population differed between the two decades studied. A higher prevalence of homozygous mutations and a lower prevalence of the heterozygous forms of the F508del mutation were observed in DII. Additionally, a higher number of compound heterozygotes, but without screening of the second mutation, was observed in DII. The failure to identify the second mutation in a large group of patients, even when the diagnosis of CF was clinically established and confirmed by two chloride level readings of >60 mEq/L, makes comparison between the two decades difficult.

Two aspects of diagnosis that should be emphasized are the use of F508del screening as the first step in *CFTR* mutation screening [42] and the need for greater attention on patients with CF diagnosed in adulthood [43] than in pediatric patients with CF [44].

### Clinical score

The SKS measures clinical severity with consideration of physical activity, radiographic findings, nutritional status, and physical examination findings [15]. This scoring system was created for children, but continues to be applied to patients with CF of all ages. Scores are undoubtedly needed for both children and adults covering all SKS characteristics. There were fewer excellent/good scores and more moderate/severe scores in DII than in DI. Similar to the results of the other analyses between these two decades, this finding can be explained because we are leading with the same population with class I, II, and III mutations whose symptoms appear early in childhood and who exhibit worsening of clinical and laboratory outcomes until the beginning of adulthood.

### CF outcome-related death

There were 18 and 31 deaths in DI and DII, respectively. There was no statistically significant difference in the number of deaths between the two decades. The increase in the number of deaths from DI to DII was proportional to the increase in the number of patients seen from DI to DII.

### Study overview

From DI to DII in our center, the management, interdisciplinary team, and conditions for CF diagnosis did not change significantly. The fewer bacterial infections and better nutrition in DII than in DI indicate the evolution and importance of early diagnosis and management of CF. These advancements provide patients with more hope and a higher quality of life. Although many differences between the two decades were related to the earlier diagnosis in DII, the changes in some variables reflect the association between the severity of CF and the aging process, such as the worsening SKS.

The implementation of IRT screening in our state was confirmed at the end of DII. In the next decade, this will likely prove to be an important milestone on par with the discovery of the *CFTR* gene in the late 1980s. Many professionals currently working with patients with CF in Brazil are participating in the implementation of the national IRT screening program. There are great expectations regarding the future benefits of early diagnosis of CF on the health of both pediatric and adult Brazilian patients. With time, new questions will emerge in the treatment and management of patients with this intriguing pathophysiological condition [45].

### Study limitations

The patients’ records provided limited information in some cases. Complete *CFTR* screening was not performed in all patients with CF enrolled in the study. In DI, it was not possible to collect information about the medications used. Only one CF referral center was included in the data, and inclusion of other centers was not possible because of the lack of information and mixed population from our state. Numerical data for spirometry, weight, height, SpO_2_, and SKS were completely recovered only for DII; thus, we were unable to perform statistical tests for numerical variables. Only one clinical score was obtained in DI.

## Conclusion

The clinical outcomes of CF changed from DI to DII in our referral center. Demographic, clinical, and laboratory analyses of patients with CF are useful and should be encouraged in referral centers worldwide. This will provide information on the clinical evolution of CF according newly available diagnostic tools, treatments, and management protocols. Furthermore, new studies on the complex features of CF will provide better knowledge of the disease. The outcomes of early diagnosis and management should be evaluated to provide a better quality of life and longer life expectancy until a personalized drug therapy is available for all *CFTR* class mutations. Each day we take steps toward better clinical outcomes for patients with CF, a pediatric disease with high mortality in some cases and an adult lung disease in other cases with better outcomes when diagnosis is performed early in life. We will continue our studies to achieve a cure for CF.

## Authors’ information

CYFIUC (Cystic Fibrosis Unicamp Center) Group: Carmen Silvia Bertuzzo, Antonio Fernando Ribeiro, Adyleia Dalbo Contrera Toro, Roberto Jose Negrao Nogueira, Gabriel Hessel, Carlos Emilio Levy, Maria Angela Gonçalves de Oliveira Ribeiro, Eulalia Sakano, Maria de Fatima Servidoni, Monica Corso Pereira, Ilma Aparecida Paschoal, Paloma Lopes Francisco Parazzi, Camila Izabel Santos Schivinski, Renata Tiemi Okuro, Luciana Cardoso Bonadia, Alfonso Eduardo Alvarez, Silvana Dalge Severino, Andressa Peixoto, Carla Cristina Souza Gomez.

## Abbreviations

CF: Cystic fibrosis
*CFTR*: Cystic fibrosis transmembrane regulator gene
IRT: Immunoreactive trypsinogen
DI: First decade
DII: Second decade
SKS: Shwachman-Kulczycki score
PI: Pancreatic insufficiency
MI: Meconium ileus
DM: Diabetes mellitus
SpO_2_: Oxygen saturation
FEV_1_: Forced expiratory volume in the first second
FVC: Forced vital capacity
OR: Odds ratio
95%CI: 95% confidence interval.
